# Clinical outcomes and biomarkers of phyllodes tumors of the breast: A single‐center retrospective study

**DOI:** 10.1002/cam4.5849

**Published:** 2023-04-20

**Authors:** Keyu Chen, Jiaojiao Xu, Wei Wang, Ruiyuan Jiang, Huanping Zhang, Xiaojia Wang, Jun Cao, Meiyu Fang

**Affiliations:** ^1^ Institute of Basic Medicine and Cancer (IBMC), Chinese Academy of Sciences, The Cancer Hospital of the University of Chinese Academy of Sciences, Zhejiang Cancer Hospital Hangzhou China; ^2^ Zhejiang Chinese Medical University Hangzhou China; ^3^ Wenzhou Medical University Wenzhou China; ^4^ Key Laboratory of Head & Neck Cancer Translational Research of Zhejiang Province, Department of rare and head and neck oncology, Institute of Cancer Research and Basic Medical Sciences of Chinese Academy of Sciences Cancer Hospital of University of Chinese Academy of Sciences, Zhejiang Cancer Hospital Hangzhou China

**Keywords:** biomarkers, distant metastases, local recurrence, outcomes, phyllodes tumor

## Abstract

**Purpose:**

Phyllodes tumors (PTs) are rare neoplasms with a certain risk of recurrence and/or metastasis. In clinical practice, there is a lack of high‐quality clinical studies and unified guidelines to guide the treatment.

**Materials and Methods:**

All malignant and recurrence/metastasis PTs were retrospectively collected, which were diagnosed from 2008 to 2022.

**Results:**

A total of 82 patients were enrolled, including 69 malignant and 13 borderline tumors. 96.3% (79/82) received surgical treatment. During a median follow‐up of 55.5 months, 20 patients (20/82, 24.4%) had distant metastasis (DM), while 32 (32/82, 39.0%) had local recurrence (LR). Univariate analysis showed the survival of PTs was associated with surgical methods (*p* < 0.001), tumor size (*p* = 0.026), and biological behavior (*p* = 0.017), but not age at diagnosis. In relapsed borderline PTs, we did not find deaths due to disease progression. Patients with DM were all malignant PTs, with disease‐progression occurring within 3 years in more than 80% of patients. Among salvage treatments, the combination of antiangiogenic drugs improved the prognosis to some extent, with a significant increase in mPFS (2.77 vs. 1.53 months), but no significant statistical results were obtained (*p* = 0.168). Lactate dehydrogenase (LDH) was an independent predictor of the prognosis for malignant PTs (*p* = 0.001, HR = 1.203, 95%CI, 1.082–1.336).

**Conclusion:**

Borderline PTs rarely metastasize, and even if LR occurs, surgical resection can lead to long‐term survival. In metastatic phyllodes tumors (MPT), systemic therapy is not effective, but antiangiogenic drugs may prolong survival. LDH is an independent prognostic factor for malignant PTs to identify high‐risk tumors.

## Graphical abstract

We retrospectively collected patients diagnosed with PTS at our institution. Eventually, 82 patients who were pathologically suggestive of malignant PTs or recurrence/metastasis PTs were enrolled. We found that borderline PTs had a relatively good prognosis even with local recurrence, whereas 25% of patients with malignant PTs progressed to advanced tumors and most died within 3 years after progression. In MPT, systemic therapy is not very effective, but antiangiogenic drugs may prolong survival. In addition, LDH may be a biological indicator for predicting the prognosis of malignant PTs.

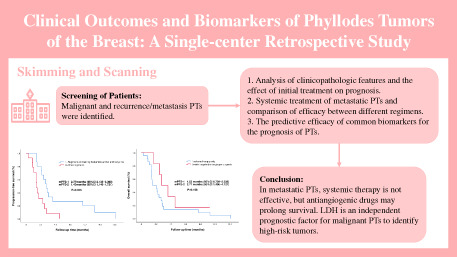

## 1 INTRODUCTION

Phyllodes tumors (PTs) are extremely rare biphasic fibroepithelial tumors, accounting for less than 1% of all breast neoplasms, most of which occur in middle‐aged women around 40 years old.^1^, ^2^ According to histological characteristics such as the degree and density of interstitial fibers, cell heterogeneity, mitosis, and tumor boundaries, PTs can be classified into benign, borderline, and malignant groups.^2^, ^3^, ^4^ The clinical feature is mostly painless breast mass. On imaging, it is extremely similar to fibroadenoma that even needle aspiration biopsy cannot accurately distinguish it.^5^, ^6^ According to the National Comprehensive Cancer Network guidelines, the preferred treatment for PTs is surgical resection.^2^, ^7^ Routine axillary lymph node dissection is not recommended.^1^, ^7^, ^8^

In biological behavior, about 60%–75% of PTs are benign, while borderline occur in 12%–26% and malignant in 10%–15%.^5^ Distant metastasis (DM) is rare in benign and borderline PTs, but there is still an approximately 25% risk of local recurrence (LR). Histologically malignant PTs are the most common type of DM, which are characterized by excessive growth of interstitial cells, marked cell atypia, and high mitotic activity.^9^, ^10^ Liposarcoma, osteosarcoma, chondrosarcoma, and other heterologous components can also appear in malignant tissue. However, regardless of their biological behavior, PTs rarely benefit from (neo) adjuvant therapy, so most patients only undergo surgical resection.^11^, ^12^, ^13^ Some patients cannot avoid LR or DM, especially malignant PTs. Once DM appears, its related treatment is extremely limited and the prognosis is very dismal. Due to the rarity of PTs, most reports are concentrated on case reports and small sample analyses, lacking the support of high‐quality data and unified guidelines.^2^, ^3^, ^14^, ^15^, ^16^, ^17^ Therefore, in the case of LR or DM, clinical oncologists will mostly follow the protocol of soft tissue sarcomas for salvage treatment.^18^, ^19^, ^20^

In this paper, a total of 82 patients with malignant PTs and recurrent/metastatic borderline PTs were treated in Zhejiang Cancer Hospital from June 1, 2008 to June 30, 2022. The purpose was to investigate the clinical characteristics, treatment efficacy, and potential biomarkers for predicting prognosis of malignant or recurrent/metastatic PT.

## 2 METHODS

### 2.1 Screening of patients

Patients diagnosed with PTs were collected from the medical records of Zhejiang Cancer Hospital. By screening, patients diagnosed with malignant or presenting with recurrence/metastasis PTs were considered subjects. Patients with benign PTs, and borderline PTs without LR or DM were excluded. Additionally, patients with other malignant neoplasms or with incomplete information were not included.

### 2.2 Clinicopathological features and biomarkers

The clinicopathological features of patients were retrospectively collected through medical records. Clinical information included age at diagnosis, menstrual status, tumor history, surgery, and adjuvant chemoradiotherapy. The pathology reports were issued by the Department of Pathology of Zhejiang Cancer Hospital or the local hospital. If there were pathological results of the same tissue from multiple hospitals, the results of our hospital should prevail. Variables extracted at initial diagnosis of PTs included tumor size, immunohistochemistry, mitotic activity, interstitial growth, and malignant heterologous components.

To better assess the patient's condition, laboratory results of blood routine examination, the hepatic and renal functions tests, and serum tumor markers were collected retrospectively from all patients on initial admission. Because this was a retrospective study, some patients were admitted with LR or DM. Therefore, we collected laboratory records of patients during the first treatment in our hospital, including before and after surgery, during recurrence, and after DM.

### 2.3 Early‐stage and advanced treatment

The treatment included the surgical method, adjuvant therapy, recurrence/metastasis time and sites, advanced treatment options, and the optimal outcome. The main treatments after disease progression are surgical resection, systemic therapy, and radiotherapy. Systemic treatment regimens were divided into containing anthracycline and ifosfamide‐based regimens, other anthracycline‐based regimens, other ifosfamide‐based regimens, platinum‐based regimens, and other systemic regimens. Treatment cycles, optimal response, recurrence/metastasis sites, and progression‐free survival (PFS) were recorded for each chemotherapy regimen. The time to progression was measured subjectively by the physician in charge, or determined by imaging examination.

### 2.4 Evaluation of the efficacy for salvage treatment

The PFS is the time from the start of the first cycle systemic therapy to progression or death or the last follow‐up. Patients who underwent surgery before and after systematic treatment were also evaluated according to the time of disease progression after treatment or last follow‐up. The overall survival (OS) is defined as the time from initial diagnosis to death for any cause or the last follow‐up. For patients without recurrence and/or metastasis, the cutoff time was the last follow‐up. According to the Response Evaluation Criteria for Solid Tumors (RECIST, version 1.1), systemic treatment response was assessed in advanced patients who received salvage therapy.

### 2.5 Last follow‐up evaluation

Date of last follow‐up was defined as the date of death or the last contact with the patient, including the medical record and telephone follow‐up. Follow‐up time was determined as the time from the diagnosis of PTs to the last follow‐up.

### 2.6 Statistical analysis

All statistical analyses were performed by statistical software SPSS (version 25.0, IBM). Continuous data were summarized descriptively using statistics, including mean, standard deviation, median, minimum, and maximum. The frequency and percentage of dichotomous data were used for descriptive summary. For time event data, including PFS and OS, the survival functions will be estimated by the Kaplan–Meier method, survival curves will be plotted, and median OS and 95% confidence intervals (95% CI) representative of the population will be provided. *p* < 0.05 was considered statistically significant.

## 3 RESULTS

### 3.1 Patient and clinicopathologic characteristics

**FIGURE 1:**
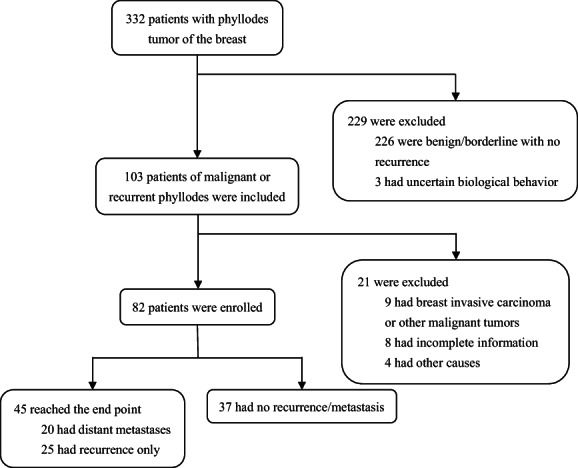
Workflow of patients screening.

A total of 332 patients with PTs were collected from the internal database, among which 103 were malignant or recurrent/metastatic PTs. Through further follow‐up, patients with incomplete information or lost to follow‐up were excluded. Finally, 82 patients were identified, with a median follow‐up time of 55.5 months (Figure 1). All patients were female. The median age at first diagnosis was 46.4 years, with the youngest under 14 years old and the oldest 73 years old.

The basic characteristics of the enrolled patients are detailed in Table 1. Fifty‐five patients (67.1%) were premenopausal at initial diagnosis. Thirty‐nine patients (47.6%) underwent mastectomy, 40 (48.8%) retained the breast, while 3 (3.7%) did not undergo surgery because of large masses or metastases. Pathologically, 69 patients (84.1%) had biological behaviors suggestive of malignancy. The median tumor size was 5 cm, with the largest tumor reaching 25 cm. Malignant heterologous components were reported in 16 patients with PTs, in which 4 (4.9%) patients had liposarcoma differentiation, 8 (9.8%) bone and/or chondrosarcoma differentiation, and 4 (4.9%) rhabdomyosarcoma differentiation.

**TABLE 1:** Basic characteristics of patients with phyllodes.

	All patients (*N* = 82)	Patients with distant metastases (*N* = 20)	Patients with local recurrence (*N* = 25)
Median follow‐up time, months (min–max)	55.5 (3.8–186.8)	18.4 (6.7–90.9)	100.2 (8.2–186.8)
Age at diagnosis of phyllodes			
Median age, years (min–max)	46.4 (13.7–72.9)	44.9 (15.7–60.3)	45.8 (13.7–59.2)
<50	57 (69.5%)	15 (75.0%)	21 (84.0%)
≥50	25 (30.5%)	5 (5.0%)	4 (16.0)
Menstrual status at diagnosis of phyllodes			
Postmenopausal	27 (32.9%)	8 (40.0%)	5 (20.0%)
Premenopausal	55 (67.1%)	12 (60.0%)	20 (80.0%)
Type of surgery for phyllodes diagnosis			
Mastectomy	39 (47.6%)	15 (75.0%)	3 (12.0%)
Breast‐conserving surgery	40 (48.8%)	3 (15.0%)	21 (84.0%)
No surgery	3 (3.7%)	2 (10.0%)	1 (4.0%)
Biological behavior of phyllodes			
Malignant	69 (84.1%)	20 (100.0%)	12 (48.0%)
Borderline	13 (15.9%)	0 (0.0%)	13 (52.0%)
Tumor size at initial diagnosis			
Median size (cm)	5.0 (1.7–25.0)	7.0 (1.8–13.0)	5.8 (2.1–25.0)
≤5 cm	36 (43.9%)	4 (20.0%)	9 (36.0%)
>5 cm	35 (42.7%)	14 (70.0%)	10 (40.0%)
Unknown	11 (13.4%)	2 (10.0%)	6 (24.0%)
Dissection of lymph nodes			
Yes	24 (29.3%)	8 (40.0%)	1 (4.0%)
No	58 (70.7%)	12 (60.0%)	24 (96.0%)
Ki‐67 index			
≤30%	19 (23.2%)	7 (35.0%)	5 (20.0%)
>30%	23 (28.0%)	9 (45.0%)	5 (20.0%)
Unknown	40 (48.8%)	4 (20.0%)	15 (60.0%)
Malignant heterologous components			
Differentiation of liposarcoma	4 (4.9%)	1 (5.0%)	0 (0.0%)
Differentiation of bone and/or chondrosarcoma	8 (9.8%)	4 (20.0%)	1 (16.7%)
Differentiation of rhabdomyosarcoma	4 (4.9%)	3 (15.0%)	1 (16.7%)
Unknown composition	66 (80.5%)	12 (60.0%)	4 (66.7%)
Adjuvant therapy			
Systemic therapy	5 (6.1%)	2 (10.0%)	1 (4.0%)
Radiation therapy	3 (3.7%)	0 (0.0%)	0 (0.0%)
No treatment	74 (90.2%)	18 (90.0%)	24 (96.0%)

### 3.2 Treatment of phyllodes after initial diagnosis

Surgery is the preferred treatment for PTs. The majority of patients (79/82, 96.3%) received surgical treatment after diagnosis. One did not undergo surgery because of systemic bone and multiple lymph node metastases at initial diagnosis, while the other two had tumors with a diameter of more than 10 cm. The latter two were minors at the time of diagnosis. Only a small proportion of patients received adjuvant systemic therapy (5/82, 6.1%) or adjuvant radiotherapy (3/82, 3.7%). Among the five patients who received systemic treatment, the tumor biological behavior was suggestive of malignancy. Four of these patients received anthracycline‐based chemotherapy (two of which included ifosfamide), while one had taxanes plus platinum. Despite systematic treatment, three patients (3/5, 60%) had recurrence and/or metastasis during the follow‐up period. The median disease‐free survival from diagnosis to recurrence and/or metastasis was 6.1 months (IQR, 3.0–51.8). The biological behavior of the three patients who received adjuvant radiotherapy was also malignant, but none of these patients had recurrence and metastasis during the follow‐up period. Thus, PTs may benefit more from radiotherapy than adjuvant chemotherapy. However, due to the small number of patients receiving postoperative adjuvant chemotherapy or radiotherapy, the reliability of this conclusion needs to be verified by more rigorous multicenter clinical studies.

### 3.3 Survival of patients with phyllodes tumor of the breast

At a median follow‐up of 55.5 months, 20 (20/82, 24.4%) had DM, 32 (32/82, 39.0%) relapsed (including seven with DM), and the remaining did not progress. Among the 82 patients, 18 patients (18/82, 22.0%) died, including 16 patients (16/18, 88.9%) with DM and two patients (2/18, 11.1%) with LR.

**FIGURE 2:**
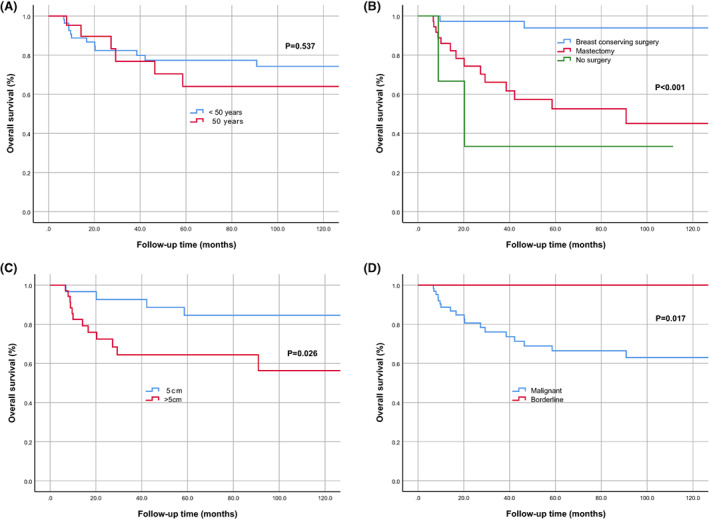
Characteristics associated with the survival of phyllodes. Overall survival (OS) curve (A) of age at diagnosis of phyllodes, (B) of the surgical method, (C) of tumor size at initial diagnosis (11 patients with unknown tumor size at initial diagnosis were excluded from the analysis), and (D) of different biological behaviors.

According to univariate analysis, the survival of PTs was related to surgical method (*p* < 0.001), tumor size (*p* = 0.026), and tumor biological behavior (*p* = 0.017), but not to age at diagnosis (*p* = 0.537) (Figure 2A–D). Patients who did not have surgery had significantly worse survival. However, in Figure 2B, the prognosis of patients who underwent breast‐conserving surgery seems to be better than that of mastectomy, which may be related to the size of PTs itself. Small tumors tend to be treated with mass resection. Analysis of tumor size revealed that larger tumors were associated with poorer survival, especially in patients with primary lesions larger than 5 cm (Figure 2C). In this study, we explored patients with malignant and recurrence/metastasis borderline PTs, so we analyzed the relationship between tumor biological behavior and prognosis. Compared with malignant PTs, borderline PTs do not affect long‐term survival even if LR or DM occurs (Figure 2D). Therefore, the treatment and prognosis of malignant PTs are of great concern, especially for patients with recurrence/metastasis of malignant tumors.

### 3.4 Clinical features of metastatic phyllodes

We further investigated 20 patients with DM, all of which were malignant (Table 1). Two patients did not receive surgical treatment due to large mass and DM, respectively. The median time from surgery or confirmed by needle biopsy to DM was 8.3 months (one patient with DM at initial diagnosis was excluded), with more than 80% progressing within 3 years of diagnosis. From these patients, lung (16/20, 80%) was the most common site of metastasis, followed by bone (7/20, 35%), brain (2/20, 10%), and other metastatic lesions (5/20, 25%). The majority of patients (15/20, 75%) had concurrent involvement of multiple sites (included lymph nodes and chest wall).

### 3.5 Treatment of metastasis phyllodes

In the condition of DM, eight patients (8/20, 40%) underwent resection (including breast/chest wall, bone, lung lesions) and four patients (4/20, 20%) received radiotherapy. Seventeen patients (17/20, 85%) received at least first‐line systemic therapy, with a total of 29 chemotherapy regimens. The remaining patients (*n* = 3) had no record of systemic treatment. It is worth noting that one patient with lung metastases had no disease progression during a follow‐up period of nearly 7 months after lobectomy. This may be related to the fact that the lung metastasis was a single lesion with a small diameter (maximum diameter 6 mm).

Of the 29 systemic regimens, the most common was regimen with ifosfamide and anthracycline (10/29, 34.5%), followed by platinum‐based regimens (4/29, 13.8%), anthracycline‐based regimens (3/29, 10.3%), and ifosfamide‐based regimens (2/29, 6.9%). Another 10 patients received other systemic treatment. Details of the various regimens are shown in Table 2.

**TABLE 2:** Systematic treatment options for patients with metastatic phyllodes (*N* = 29).

Systemic chemotherapy regimens	Number of patients (*N* = 29)
With ifosfamide and anthracycline (10)	
Ifosfamide/anthracycline	6
Ifosfamide/anthracycline/anlotinib	2
Ifosfamide/anthracycline/anlotinib/dacarbazine	1
Pirarubicin/cyclophosphamide/vindesine and etoposide/ifosfamide alternately	1
Other ifosfamide‐based regimens (2)	
Ifosfamide/dacarbazine	2
Other anthracycline‐based regimens (3)	
Anthracycline alone	2
Anthracycline/cyclophosphamide	1
Platinum‐based regimens (4)	
Cisplatin/temozolomide	1
Cisplatin/abraxane/apatinib	1
Carboplatin/gemcitabine	1
Carboplatin/irinotecan	1
Other systemic regimens (10)	
Gemcitabine/thalidomide	2
Gemcitabine/abraxane/anlotinib	1
Capecitabine/docetaxel	1
Capecitabine/endostar	1
Vinorelbine/bevacizumab/tislelizumab	1
Eribulin	1
Abraxane	1
Anlotinib	1
Temozolomide	1

*Note*: Due to the small sample size of this retrospective study, none of the systematic treatment regimens took into account the number of lines of the regimen and the effect of front‐line treatment on the efficacy of back‐line regimens.

### 3.6 The PFS of metastatic phyllodes after systemic treatment

**FIGURE 3:**
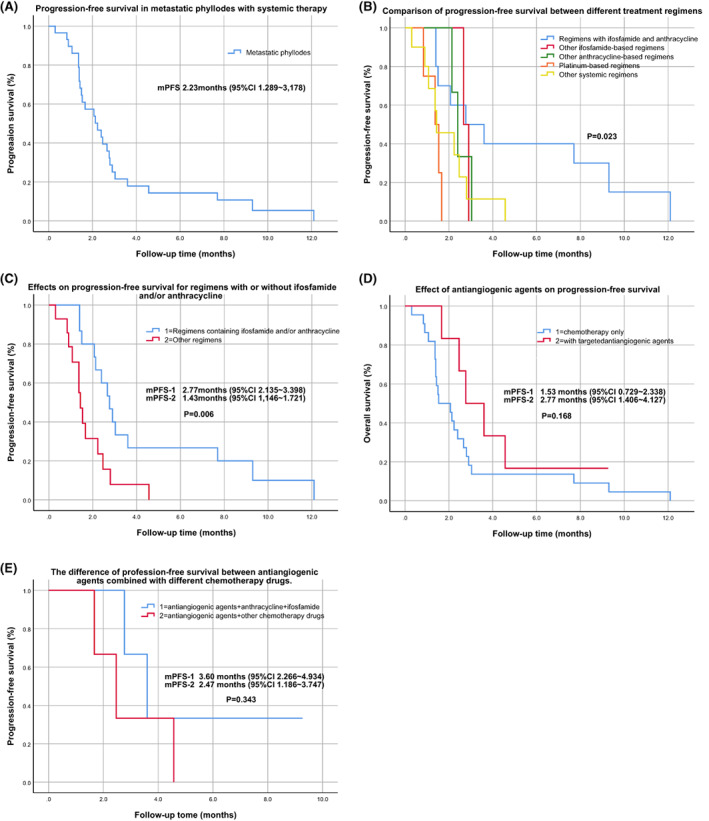
Survival analysis of metastatic phyllodes. (A) The progression‐free survival (PFS) in metastatic phyllodes with systemic therapy. (B) Comparison of the PFS between different treatment regimens. (C) Effects on PFS for regimens with or without ifosfamide and/or anthracycline. (D) Effect of antiangiogenic agents on PFS. (E) The difference of PFS between antiangiogenic agents combined with different chemotherapy drugs.

As this retrospective study was affected by a small sample, we calculated the median PFS (mPFS) for all systemic treatments, regardless of the number of lines over the course of disease and the effect of front‐line treatment on the efficacy of the back‐line regimens. The mPFS was 2.23 months (95% CI, 1.289–3.178) (Figure 3A). We divided all systematic treatments regimens into five categories. Among the five different types of chemotherapy, regimens with ifosfamide and anthracycline were the most effective, with mPFS of 2.77 months (95% CI, 0.391–5.143), followed by ifosfamide‐ or anthracycline‐based regimens, with mPFS of 2.67 and 2.40 months, respectively. The efficacy of platinum‐based regimens and other regimens was not promising, with mPFS lasting less than 1.5 months (1.37 and 1.43 months). The majority of patients had disease progression after the first cycle (Figure 3B). Based on this, we reclassified chemotherapy types into regimens containing ifosfamide and/or anthracycline and other regimens. As shown in Figure 3C, regimens containing ifosfamide and/or anthracycline (mPFS 2.77 months, 95% CI, 2.135–3.398) had more significant benefits than other systemic regimens (mPFS 1.43 months, 95% CI, 1.146–1.721), with nearly onefold longer mPFS (*p* = 0.006).

In the 29 systemic regimens, seven patients (7/29, 24.1%) were combined with antiangiogenic agents. Survival analysis with or without antiangiogenic agents indicated that patients with combined drugs improved the prognosis to some extent, with mPFS nearly doubling compared with those without targeted drugs (2.77 vs. 1.53 months). Unfortunately, from the perspective of statistical analysis, there was no significant difference between the two groups (*p* = 0.168), but according to the analysis trend in Figure 3D, the combined group manifested a better treatment effect, which may be caused by the insufficient number of patients with combined medication. The specific characteristics of seven patients receiving antiangiogenic agents were recorded minutely in Table 3. Notably, the combined antiangiogenic regimens resulted in an overall disease control rate (DCR) of 71.4%, with 42.9% achieving partial response (PR) by ifosfamide, anthracycline, and anlotinib. In these seven patients, although statistically no significant difference was shown (*p* = 0.343), from the perspective of survival (Figure 3E), the combination of the three drugs seems to have synergistic antitumor effect, and the efficacy is better than that of the combination of other chemotherapy drugs (3.60 vs. 2.47 months).

**TABLE 3:** Basic characteristics and efficacy in patients with targeted antiangiogenic drugs (*N* = 7).

Patients	Age at diagnosis	DFS (months)	Distant metastasis sites	Systemic treatment regimen	Optimal effect	PFS (months)	OS (months)	Survival
Case 1 (LLY)	43	4.4	Lung, chest wall	Cisplatin/abraxane/**apatinib**	PD	1.7	6.9	Dead
Case 2 (JMY)	57	2.5	Lung, chest wall, axillary lymph nodes	Ifosfamide/dacarbazine/liposome doxorubicin/**anlotinib**	PR	2.8	7.9	Dead
Case 3 (LYX)	18	5.3	Lung, breast masses	**Anlotinib** only	PD	2.5	20.1	Alive
Case 4 (LYX)	18	5.3	Lung	Gemcitabine/abraxane/**anlotinib**	SD	4.6	20.1	Alive
Case 5 (LYX)	18	5.3	Lung	Vinorelbine/**bevacizumab**/tislelizumab	SD	NA	20.1	Alive
Case 6 (XGW)	53	53.0	Lung, bones, brain	Epirubicin/ifosfamide/**anlotinib**	PR	3.6	58.6	Dead
Case 7 (MHL)	41	15.7	Lung	Ifosfamide/liposome doxorubicin, **anlotinib** maintained	PR	9.3	26.6	Alive

*Note*: Antiangiogenic drugs include apatinib, anlotinib, and bevacizumab, all of which are indicated in bold in the table. Among them, apatinib and anlotinib are both small molecular tyrosine kinase inhibitors independently developed in China. The former targets vascular endothelial growth factor‐2 (VEGFR‐2), while the latter can effectively inhibit VEGFR, PDGFR, FGFR and other multi‐target kinases. Cases 3, 4, and 5 were the same patient, but antiangiogenic drugs were used in different late‐stage treatment lines.

Abbreviations: DFS, disease‐free survival; PD, progressive disease; PFS, progression‐free survival; PR, partial response; SD, stable disease.

### 3.7 Comparison of efficacy of different treatment regimens

The efficacy of systemic therapy for metastatic phyllodes tumors (MPT) could be assessed for optimal outcome in 27 of 29 episodes. The other two patients were not evaluated because of evaluable lesion resection or unclear efficacy. We did not find a complete response to 27 systemic treatments that were evaluable. Of these treatments, 5 (18.5%) had PR and 6 (22.2%) had stable disease, with a DCR of 40.7% (Table 4). Only ifosfamide and anthracycline regimens resulted in PR, while most had disease progression soon after treatment.

**TABLE 4:** Optimal efficacy of different treatment regimens (*N* = 27).

Systemic chemotherapy regimens	PR (%)	SD (%)	PD (%)	DCR (%)
With ifosfamide and anthracycline	5 (55.6)	1 (11.1)	3 (33.3)	6 (66.7)
Other ifosfamide‐based regimens	0 (0)	0 (0)	1 (100)	0 (0)
Other anthracycline‐based regimens	0 (0)	2 (66.7)	1 (33.3)	2 (66.7)
Platinum‐based regimens	0 (0)	0 (0)	4 (100)	0 (0)
Other systemic regimens	0 (0)	3 (30.0)	7 (70.0)	3 (30.0)
All regimens	5 (18.5)	6 (22.2)	16 (59.3)	11 (40.7)

*Note*: Two patients were not evaluated for optimal outcome because of evaluable lesion resection or unclear efficacy.

Abbreviations: DCR, disease control rate; PD, progressive disease; PR, partial response; SD, stable disease.

### 3.8 The OS of metastatic phyllodes

Once DM occurred, the survival of patients significantly decreased, with nearly 80% dying within 5 years. In the 20 patients with DM we included, the mOS was only 20.3 months (95% CI, 5.792–34.875). Four patients were still alive at the last follow‐up, of which three continued to receive treatment or follow‐up monitoring, and the other patient was lost to follow‐up after local surgical curettage of bone metastases.

### 3.9 Clinical features and treatment of recurrent phyllodes

Another 25 patients with only LR were analyzed, with biological behaviors including malignant (12/25, 48%), and borderline (13/25, 52%). More than 50% of patients with recurrence occurred within 1 year after the initial surgery, most of which were ipsilateral breast mass (23/25, 92%), chest wall (8/25, 32%), and ipsilateral axillary lymph node/mass (5/25, 20%). Twenty‐three patients (23/25, 92%) underwent surgery at least once. Of the relapsed patients we included, only two patients died at 1 and 3 years due to repeated chest wall relapses and treatment abandonment. Compared with MPT, patients with LR, especially borderline tumors, can basically achieve long‐term tumor‐free survival as long as they receive standard treatment.

### 3.10 Biomarkers associated with the prognosis of malignant phyllodes

From the above analysis, it can be seen that although PTs have a certain risk of recurrence, the prognosis is relatively excellent, except for MPT. Since all MPT were malignant PTs, biomarkers of 69 patients with malignant PTs were analyzed to find characteristic prognostic indicators.

Univariate Cox regression analysis showed that carbohydrate antigen 153 (CA153), carbohydrate antigen 125 (CA125), lactate dehydrogenase (LDH), and neutrophil and lymphocyte ratio (NLR) were all risk factors for the prognosis of malignant PTs. In multivariate regression analysis, LDH was considered to be the only independent predictor associated with the prognosis of malignant PTs (*p* = 0.001, HR = 1.203, 95% CI, 1.082–1.336) (Table 5). It can be seen that patients with increased LDH are more prone to DM, which affects survival.

**TABLE 5:** Predictive biomarker for malignant phyllodes tumors.

	Univariate Cox regression			Multivariate Cox regression		
	*B*	*p* value	HR (95% CI)	*B*	*p* value	HR (95% CI)
CA153	0.126	0.041	1.134 (1.005–1.279)	0.030	0.635	1.030 (0.910–1.166)
CEA	0.298	0.360	0.743 (0.392–1.405)			
CA125/10	0.085	0.023	1.089 (1.012–1.172)	0.070	0.353	0.932 (0.803–1.081)
LDH/10	0.156	<0.001	1.169 (1.095–1.249)	0.184	0.001	1.203 (1.082–1.336)
NLR	0.138	0.002	1.148 (1.051–1.253)	0.020	0.821	1.021 (0.856–1.217)
LMR	0.247	0.063	0.781 (0.602–1.014)			

Abbreviations: CA125, carbohydrate antigen 125; CA153, carbohydrate antigen 153; CEA, carcinoembryonic antigen; LDH, lactate dehydrogenase; LMR, lymphocyte and monocyte ratio; NLR, neutrophil and lymphocyte ratio.

## 4 DISCUSSION

PTs, also known as phyllodes cystosarcoma, was first described and named by Müller in 1838, which were initially considered to be benign tumors. The nomenclature was not officially given until 2003 by the World Health Organization.^4^ Because of its rarity and bidirectional biological behavior, clinicians largely rely on clinical experience and case reports for treatment. This study supplemented the reported data of PTs to a certain extent, focusing on the clinicopathological features and prognostic factors of malignant PTs.

Of the 69 patients with malignant PTs included, 20 (29.0%) eventually developed MPT, which appeared to be slightly higher than the previously reported risk.^3^, ^14^ This may be related to attributes of the research center and referral bias. In our sample, there were no patients with MPT who were initially diagnosed as benign or borderline tumors. This result was basically consistent with the results reported in previous studies, namely, DM were seen in malignant PTs, but were extremely rare in benign and borderline tumors.^14^, ^15^ In distant metastatic lesions, lung, bone, and brain metastases are most commonly involved, usually accompanied by other metastases (including LR and other DM sites).^14^, ^15^, ^17^ Here, 40% had multiple DM and 25% had only one metastasis, which contradicted the results of the study by Parkes et al., in which the metastases were mainly single lesions.^14^, ^15^

The time from the diagnosis of primary lesion to the appearance of DM varies widely, with most occurring within 3 years of initial discovery.^14^, ^17^, ^21^, ^22^, ^23^ In our group, the median time was 8.3 months. The patient with the shortest disease‐free survival developed metastases in chest wall, axillary lymph nodes, and bilateral lungs within 2 months after total mastectomy.

DM inevitably requires systemic treatment.^14^, ^15^, ^17^ However, many researchers have shown low efficacy of chemotherapy.^14^, ^17^ Even when multiple chemotherapeutic drugs are combined, the best effect is only to cause tumor regression in a short period.^14^, ^24^, ^25^ Among these protocols, the regimen with ifosfamide and anthracycline was considered to be the one with the best tumor control ability.^14^, ^15^, ^17^ In patients in our study, although this regimen still had the best effect, the mPFS was only 2.77 months, the longest was 12.1 months, and the best effect was only PR. Unlike ours, this regimen in European and American populations appears to be more effective than ours. In Ratan's retrospective analysis of systemic therapy for MPT, the AI regimen resulted in a PFS of 9.10 months, and even the least effective regimen had a PFS of 1.67 months.^15^ In a multicenter retrospective study by Palassini, the mPFS of AI reached 5.7 months, and nearly 45% of the patients had tumor response.^17^ These may be related to racial, environmental, and genetic differences.

Noteworthy, some of the patients received antiangiogenic agents. The combination of targeted drugs nearly doubled mPFS compared with no targeted drugs. Although the differences were not statistically significant, the survival curves indicated a greater benefit with the combination than without antiangiogenic agents. Moreover, the efficacy of antiangiogenic agents combined with ifosfamide and anthracycline is better than with other chemotherapy drugs, which is inseparable from the synergistic antitumor effect of the three drugs. However, in advanced treatment, only seven received targeted drugs, a key factor in the lack of a statistically significant difference. To our knowledge, this finding has not been previously reported in the treatment of any MPT. Therefore, we believe that clinical trials of combined antiangiogenic agents can be considered at the later stage to further verify the efficacy, especially the benefit of antiangiogenic agents combined with ifosfamide and anthracycline.

Peripheral blood biomarkers have shown unique clinical value in the early diagnosis and prognosis prediction of a variety of tumor and non‐tumor diseases.^26^ LDH, as one of the important enzymes in glucose metabolism, provides energy for cell proliferation and metabolism. Previous studies have found that LDH activity is often increased in pleural and abdominal effusion caused by malignant tumor metastasis, which is associated with poor clinical prognosis and drug resistance.^27^, ^28^, ^29^ Here, we first explored that the level of LDH in peripheral blood was closely associated with the metastasis of malignant PTs. Additionally, CA153, CA125, and NLR have certain value in predicting the prognosis of patients with malignant PTs. Since there is no additional limit on the collection time of peripheral blood indicators, they can only be used as reference factors to evaluate tumor prognosis. Additionally, this is only a single‐center surveillance data, so they need to be verified by a multiagency collaboration. In the future, these molecular signatures of rare breast tumors may be widely used to identify high‐risk tumors.

Moreover, some studies have tried to search more specific indicators from immunohistochemical markers to predict the risk of metastasis, but none of them have been clinically validated.^30^, ^31^, ^32^, ^33^, ^34^ However, a part of studies has reported that the occurrence of PTs is associated with genetic mutations of RB1, BRCA1, TP53, and other genes.^35^, ^36^, ^37^, ^38^, ^39^, ^40^ In the association between germline mutations and carcinogenesis, Birch and his colleagues found a strong correlation between patients carrying germline TP53 mutations and the development of malignant PTs.^41^ Compared with the general population, patients with germline TP53 mutations had the greatest increase in PTs.^41^ Subbiah reported a malignant phyllodes patient with PI3K/Akt/mTOR‐activated NRAS mutation that may be responsive to taxanes, especially albumin‐bound paclitaxel.^42^ These findings undoubtedly provide a therapeutic target for this rare and deadly disease, which may lead to the development of new therapeutic modalities in this disease. However, only one of the patients we enrolled could be followed up with plasma circulating tumor DNA. Unfortunately, no variation was detected in BRCA1/2, ERBB2, and PIK3CA genes.

Overall, this was only a single‐center retrospective study with a limited number of patients with LR and DM. Only a small number of patients received salvage therapy, and the response was not satisfactory. Therefore, this rare tumor still needs multi‐institutional cooperative, prospective studies to improve the salvage treatment decisions in the advanced stage, in order to enhance the quality of life and prolong survival.

## 5 CONCLUSION

In conclusion, the prognosis of borderline PTs with LR is relatively good, which can even be cured as long as the treatment is standardized. However, 25% of patients with malignant PTs will progress to advanced tumors. The efficacy of salvage chemotherapy is extremely limited, while the duration of the response is short. Most patients die within 3 years after progression. Our study is the first to show the effect of LDH in predicting prognosis. We believe that with the progress of research, biomarker detection will become the future development trend of this rare and poor prognosis tumor, and provide new clues to identify effective treatment methods. In addition, international collaboration in clinical trials is necessary for rare tumors.

## AUTHOR CONTRIBUTIONS

**Keyu Chen:** Formal analysis (equal); investigation (equal); writing – original draft (equal). **Jiaojiao Xu:** Formal analysis (equal); writing – original draft (equal). **Wei Wang:** Formal analysis (equal); investigation (equal). **Ruiyuan Jiang:** Formal analysis (equal); methodology (equal). **Huanping Zhang:** Formal analysis (equal); investigation (equal). **Xiaojia Wang:** Conceptualization (equal); supervision (equal). **Jun Cao:** Conceptualization (equal); funding acquisition (equal); resources (equal); supervision (equal); writing – review and editing (equal). **Meiyu Fang:** Conceptualization (equal); funding acquisition (equal); methodology (equal); supervision (equal); writing – review and editing (equal).

## FUNDING INFORMATION

This study was supported by the National Natural Science Foundation of China (81702653 to J.C.).

## CONFLICT OF INTEREST STATEMENT

The authors declare that there is no conflict of interest regarding the publication of this paper.

## ETHICS STATEMENT

This retrospective study involving human participants was in accordance with the ethical standards of the institutional and national research committee and with the 1964 Helsinki Declaration and its later amendments or comparable ethical standards. The Human Investigation Committee (IRB) of Zhejiang Cancer Hospital approved this study.

## PATIENT CONSENT STATEMENT

All patients and their families were informed and agreed to participate in the retrospective study.

## Data Availability

The datasets generated and analyzed during the current study are not publicly available due to contain patient's privacy, but are available from the corresponding author on reasonable request.
